# Microencapsulation of *Bacillus velezensis* Using Alginate-Gum Polymers Enriched with TiO_2_ and SiO_2_ Nanoparticles

**DOI:** 10.3390/mi13091423

**Published:** 2022-08-29

**Authors:** Mojde Moradi Pour, Roohallah Saberi Riseh, Reza Ranjbar-Karimi, Mohadeseh Hassanisaadi, Abbas Rahdar, Francesco Baino

**Affiliations:** 1 Department of Plant Protection, Faculty of Agriculture, Vali-e-Asr University of Rafsanjan, Rafsanjan 7718897111, Iran; 2 Department of Chemistry, Faculty of Science, Vali-e-Asr University, Rafsanjan 7718893514, Iran; 3 Department of Plant Protection, Faculty of Agriculture, Shahid Bahinar University of Kerman, Kerman 7618411764, Iran; 4 Department of Physics, University of Zabol, Zabol 9861335856, Iran; 5 Institute of Materials Physics and Engineering, Department of Applied Science and Technology, Politecnico di Torino, 10129 Turin, Italy

**Keywords:** biological control, biological, plant diseases, polymer, natural gums, whey protein, nanoparticles

## Abstract

Bacillus bacteria are a group of plant growth stimulants that increase plant growth and resistance to plant pathogens by producing various metabolites. With their large surface area and small size, nanoparticles can be used in controlled-release formulations and increase the efficiency of the desired product. Encapsulation of biological agents in combination with nanoparticles can be an essential step in increasing the performance of these agents in adverse environmental conditions. In this study, which is the result of a collaboration between scientists from Italy and Iran, *Bacillus velezensis* was encapsulated in alginate combined with whey protein and zedo, mastic, and tragacanth gums in the presence of silica and titania nanoparticles to obtain two-layer and multilayer assemblies acting as novel, smart micro-encapsulation systems. The results of laboratory studies showed that the *B. velezensis* could produce protease, lipase, siderophore, auxin, and a dissolution of mineral phosphate. Scanning electron microscopy images (SEM) showed that the studied microcapsules were almost spherical. Moisture affinity, swelling, and efficiency of each microcapsule were examined. The results showed that the highest encapsulation efficiency (94.3%) was related to the multilayer formulation of alginate-whey protein-zedo. XRD and FTIR spectroscopy showed that the alginate, whey protein, and zedo were mixed properly and no incompatible composition occurred in the reaction. This study aimed to provide a suitable formulation of biofertilizers based on biodegradable compounds as an alternative to chemical fertilizers, which is low cost and very effective without harming humans and the environment.

## 1. Introduction

Human life is completely dependent on plants, so much so that 90% of the nutrients needed by humans come from plants; hence, any factor that disrupts the production of agricultural products will directly affect human nutritional needs. Pests, weeds, and plant diseases can be mentioned among the main factors that can reduce the production of agricultural products. Among these, plant diseases are very important due to the rapid and sudden damage they cause [1]. The most effective way to prevent plant diseases is to use chemical toxins. However, this approach has some disadvantages such as harm to other living organisms and a reduction in beneficial soil microorganisms. Biological control is a promising strategy for the replacement of chemical compounds and an alternative to long-term sustainability and effective management of soil pathogens [2]. Bacillus bacteria as biological control agents (BCAs) have been considered for many years [3]. Most pathogenic fungi are inactivated or suppressed by volatile and non-volatile extracellular substances produced by most Bacillus species [4]. These bacteria produce plant cell wall-degrading enzymes (e.g., chitinase, cellulase, and β-glucanase), antibiotics, and plant growth-promoting hormones, increasing nutrient uptake and inducing plant resistance against plant pathogens, thus yielding an overall enhancement of plant growth [5]. In biological control strategies, there is high interest in isolates that produce a broad spectrum of antimicrobial metabolites and can simultaneously control more than one disease in a particular plant host, and have mechanisms to enhance growth and induce resistance in the plant. These isolates are worthwhile for production and commercial applications (fermentation, formulation, and packaging). In conventional formulations of BCAs, the population of biocontrol bacteria may be negatively affected by environmental fluctuations such as temperature, competition, pH, salinity, and drought [6]. Therefore, the application of BCAs in novel and valuable formulations which increase their stability and efficacy is critical. In this regard, the use of encapsulation technology extends the shelf-life of biocontrol bacteria during production and storage, making them more durable in the soil, and, thus, improving their performance by controlled release [7]. Encapsulation, as one of the newest methods to cover the cells of microorganisms with a layer of hydrocolloids on a microscopic scale in order to entrap and separate them from the environment [8,9], results in the survival of probiotic bacteria during targeted storage and release [10]. Regarding encapsulation, biopolymers are low-cost, biodegradable, and environmentally friendly options having widespread use as carriers [11,12]. Among them, sodium alginate is a polysaccharide abundant in the cell wall of brown algae such as *Macrocystis pyrifera*, *Ascophyllum nodosum*, *Sargassum sinicola*, and bacteria species such as Pseudomonas and Azotobacter [13]. Alginate (Alg) has several industrial applications, including enhancing the viscosity of gels, storing and transferring various biomolecules, and preserving water [14]. Numerous studies have been performed on the use of alginate in the control of plant pests and diseases. It is proven that the use of alginate in encapsulation of BCAs causes an efficient control of *Rhizoctonia solani* on beans [15], *Fusarium solani* on potatoes [16], and *R. solani* on potatoes [17] and has a significant role in enhancing the growth-related factors of the plant. However, alginate has some disadvantages in encapsulation. For example, the porosity of alginate gel makes the microcapsules nonstable and permeable to moisture, resulting in reduced protection against environmental factors. Therefore, it is recommended to use some materials as fillers for the alginate pores [18]. For this purpose, proteins and gums can be offered as a suitable choice; specifically, whey protein (WPC) and Iranian native gums are very valuable. Whey is a by-product of large-scale cheese factories, most of which is not used and causes serious contamination and the problem of waste disposal [19]. Among gums, we can name tragacanth gum (TG), zedo gum (ZG), and mastic gum (MG). Tragacanth is a natural gum derived from the *astragalus* plant [20]. This plant grows mostly in Southwest Asia. Iran is one of the most important producers of TG in the world. Zedo gum is secreted from the plant (*Amygdalus scoparia*) of the almond species. Increasing the concentration of this gum in the oil-in-water emulsion increases the formation capacity and stability of the emulsion [21]. Wild pistachio, with the scientific name *Pistacia atlantica,* is the source of mastic gum. It is a very light, thick, and very sticky green gum. Iran has a plentiful supply of polysaccharide compounds such as the gums described above due to the richness of plant resources and, thus, the use of these gums is common in various sciences. The use of Iranian native gums has also been encouraged due to the importance of hydrocolloids in many industrial processes and the high price of these compounds when imported from abroad. Indeed, extraction and industrial consumption of native hydrocolloid compounds such as gums can have an important role in the country’s economic situation. Therefore, the use of gums derived from natural elements can be a creative, cost-effective, and eco-friendly idea for the development of capsules loading BCAs. In addition to the use of gum and biopolymers for the production of microcapsules, the application of emerging technologies such as nanotechnology can provide further benefits. Today, nanotechnology has entered the various sciences and has led to the use of nanoparticles (NPs) in a myriad of fields, including agricultural science [22,23]. The use of silica nanoparticles (SiO_2_ NPs) increases the resistance of plants to stress, pests, and plant diseases [24]. Titania nanoparticles (TiO_2_ NPs) have also been recently introduced in agricultural sciences as they cause microbial agents to adhere to plant roots, so it can be expected that their use in biological fertilizers will increase the percentage of microbial agent colonization on roots [25]. In recent years, a novel microencapsulation technology based on the layer-by-layer assembly of oppositely charged polymers has been developed. The multilayer microcapsules can also be modified with the addition of other polymers (lipids, carbohydrates, proteins, polymeric nanoparticles, and bioactive compounds) to provide the microcapsules with special functions [26].

Considering that antagonistic bacteria are sensitive to environmental conditions, such as competition, pH fluctuations, temperature changes, etc., and can be therefore quickly destroyed before colonization, the purpose of this research is to use coatings for encapsulation of biocontrol bacteria with nanoparticles and natural gums, which, while protecting bacteria, increase the growth and resistance of the plant against stresses.

In the present work, we report the encapsulation of BCAs with alginate combined with whey protein and zedo, mastic, and tragacanth gums in the presence of silica and titania nanoparticles in layer-by-layer/multilayer assemblies against take-all disease. Despite the progressive growth of reports on the utilization of BCAs for suppressing phytopathogens, there is not yet an efficient and appropriate formulation for the application of BCAs in sustainable agriculture. So, in order to minimize such a gap, this work aimed to (1) develop microcapsules by a layer-by-layer/multilayer approach using alginate combined with whey protein and zedo, mastic, and tragacanth gums enriched with silica and titania NPs; (2) evaluate the efficiency of these microcapsules in the loading of *B. velezensis*; (3) characterize the microcapsules by SEM, FTIR, and XRD; and (4) assess the efficiency *Bacillus velezensis* loaded in the microcapsules to control the take-all disease caused by *Gaeumannomyces graminis* var. *tritici*.

## 2. Materials and Methods

### 2.1. Preparation of Antagonist Bacteria and Pathogen

In this study, *Bacillus velezensis* and *Gaeumannomyces graminis* var. *tritici* were obtained from the biological control collection and the mycology collection, Department of plant protection, Faculty of Agriculture, Vali-e-Asr University, Iran. Bacteria were cultured in Nutrient Broth (NB, Merck, Darmstadt, Germany) medium. A pure culture of *B. velezensis* was stored in distilled water for the short term. For long-term storage, a 24-h liquid culture of bacteria in the NB medium was mixed with 40% sterile glycerol in Eppendorf vials and refrigerated at −80 °C [27]. Subsequently, *Gaeumannomyces graminis* var. *tritici* was cultured on the potato dextrose agar (PDA, Merk, Darmstadt, Germany), incubated at 29 °C, and stored as a pure culture at 4 °C before use.

### 2.2. In Vitro Evaluation of the Antifungal Activity of Bacillus velezensis against Gaeumannomyces graminis var. tritici

In order to evaluate the in vitro antifungal activity of *B. velezensis* against *Gaeumannomyces graminis* var. *tritici*, a cross-culture bioassay was assessed in a PDA medium as described by Thomashow and Waller [28]. Briefly, a loop of fresh bacterial culture was cultured at a point half a centimeter from the edge of the Petri plate and kept at 28 °C for 48 h. A mycelial disc of the fresh culture of the fungus was placed in the center of the Petri plate containing the bacteria, and the Petri plates were incubated at 28 °C for 72 h. Control treatments received sterile distilled water in the same manner. This experiment was performed in three replications, and the growth rate of fungal mycelium was recorded up to the margin of the bacterial colony.

### 2.3. Evaluation of Bacillus velezensis to Produce Antifungal and PGPR Metabolites

#### 2.3.1. Protease Enzyme

The ability of *B. velezensis* to produce the protease enzyme was assessed according to Maurhofer et al. [29]. After five days on the skim milk agar medium, the formation of a clear halo around the bacterial colony indicates the ability to produce the protease enzyme.

#### 2.3.2. Lipase Enzyme

Lipase activity was evaluated in a medium containing Tween 80 [30]. After one week, the production of a cloudy halo or sedimentary spots around the bacterial colony indicates the hydrolysis of lipids by *B. velezensis*.

#### 2.3.3. Indole-3-Acetic Acid

The auxin synthesis ability of *B. velezensis* was evaluated using the method described by Patten and Glick [31]. In order to evaluate the auxin production ability of the bacterial strain, bacteria were firstly cultured in tryptic soy broth (TSB, 2.5 g dextrose, 2.5 g di-potassium hydrogen phosphate, 3 g peptone, 17 g tryptone, and 5 g sodium chloride) for 48 h. 100 μL of the bacteria suspension was transferred to 25 mL of DF (Dworkin and Foster) salt minimal medium (4 g KH_2_PO_4_, 6 g Na_2_HPO_4_, 0.2 g MgSO_4_·7H_2_O, 1 mg FeSO_4_·7H_2_O, 10 µg H_3_BO_3_, 10 µg MnSO_4_, 70 µg ZnSO_4_, 50 µg CuSO_4_, 10 µg MoO_3_, 2 g glucose, 2 g gluconic acid, 2 g citric acid, 12 g agar (for solid media), and 1000 mL distilled water) containing 0, 50, 100, and 200 mg/L tryptophan, a precursor to auxin production. After 48 h of incubation at 28 °C, the bacterial suspension was centrifuged at 1000 rpm for 15 min. Then, 1 mL of the above solution was mixed with 4 mL of Salkowaki reagent. After 20 min, its light absorption was read using a spectrophotometer at 535 nm.

#### 2.3.4. Siderophore

*B. velezensis* was assessed to synthesize siderophore according to the method described by Alexander and Zuberer [32].

#### 2.3.5. Evaluation of the Phosphate Mineral Solubility

In order to evaluate the phosphate solubility of *B. velezensis*, a culture medium containing 1.0% (*w*/*v*) glucose, 0.05% (*w*/*v*) (NH_4_)_2_SO_4_, 0.02% (*w*/*v*) NaCl, 0.02% (*w*/*v*) KCl, 0.01% (*w*/*v*) CaCl_2_·2H_2_O, 0.01% (*w*/*v*) MgSO_4_·7H_2_O, 0.05% (*w*/*v*) MnSO_4_·7H_2_O, 0.05% (*w*/*v*) FeSO_4_·7H_2_O, 0.05% (*w*/*v*) yeast extract, and 0.5% (*w*/*v*) Ca_3_(PO_4_)_2_ in distilled water (pH 7.5). Ca_3_(PO_4_)_2_ was autoclaved first. Then, the other sterile ingredients were aseptically mixed after autoclaving. The bacteria were cultured in a spot on the center of the culture medium and incubated at 28 °C. After 48 h, the diameter of a clear halo around the bacterial colony, indicating the solubility of phosphate around the bacterial colony, was measured.

### 2.4. Preparation of Bacterial Microcapsules and Study of Their Properties

#### 2.4.1. Materials Utilized in Microcapsule Production

Sodium alginate and CaCl_2_ were obtained from the Merck Company. The whey protein was prepared by the Sigma Company (Taufkirchen, Germay). Used natural gums were collected manually. The SiO_2_ and TiO_2_ nanoparticles were synthesized in the Nanotechnology Laboratory of Vali-e-Asr University of Rafsanjan (Rafsanjan, Iran).

#### 2.4.2. Synthesis of TiO_2_ NPs

The synthesis of titania nanoparticles (TiO_2_ NPs) was performed based on the method described by Nagaraju et al. [33]. Briefly, 0.4 g of TiO_2_ (Merck; particle size about 1–4 mm, density = 4.26 g/cm^3^) was added to 5 mL the hydrogen peroxide (H_2_O_2_) and stirred gently. Then, 35 mL of H_2_O_2_, 2 mL of HNO_3_, and butyl-methyl imidazolium chloride (0.5 g) were added slowly and stirred continuously. This solution was subjected to hydrothermal treatment in an autoclave for two days at 130 °C. After two days, the autoclave was cooled to room temperature. After centrifuging the product, it was washed repeatedly with water and ethanol. Then, the product was mixed with acetonitrile and stirred for 24 h to remove the ionic liquid. The final product was centrifuged and dried in a vacuum oven at 80 °C. The following equation presents the TiO_2_ NPs formation mechanism (Equation (1)):

(1) $$\begin{array}{l} \mathrm{Ti}{\left(\mathrm{OR}\right)}_{4}\overset{\begin{array}{c} \mathrm{Acetylatone}\\ \mathrm{Ethanol}\\ \mathrm{HCL} \end{array}}{\to }\overset{\mathrm{Hydrolysis}\;}{\to }{\;\mathrm{TiO}}_{2}\left(\mathrm{Sol}\right)\\ \to O-\mathrm{Ti}-O-\mathrm{Ti}-O-\mathrm{Ti}-O-\mathrm{Ti}-O-\mathrm{Ti}-O\;\left(\mathrm{Polymer}\;\mathrm{gel}\right)\overset{\Delta }{\to }{\mathrm{TiO}}_{2}+H_{2}O \end{array}$$

where Ti(OR)_4_ = tetraethyl orthotitanate.

#### 2.4.3. Synthesis of SiO_2_ NPs

The synthesis of silica nanoparticles was performed based on the method described by Zulfiqar et al. [34]. In brief, a mixture of ammonia and ethanol was prepared at a 1:3 ratio. A sodium silicate solution was added dropwise to the ammonia and ethanol mixture and was placed on the stirrer for one hour. After washing the product, it was centrifuged and dried in a vacuum oven to obtain silica nanoparticles. The following equation presents the SiO_2_ NPs formation mechanism (Equation (2)):

(2) $$\mathrm{Si}{\left(\mathrm{OR}\right)}_{4}\overset{\begin{array}{c} \mathrm{Methanol}\\ \mathrm{Ethylenediamine} \end{array}}{\to }\;\mathrm{Si}{\left(\mathrm{OH}\right)}_{4}\overset{\Delta }{\to }{\;\mathrm{SiO}}_{2}+H_{2}O$$

where Ti(OR)_4_ = tetraethyl orthosilicate.

#### 2.4.4. Characterization of NPs

The scanning electron microscope (SEM) technique was performed to evaluate the morphology and topography of synthesized NPs by scanning the surface of the NPs. XRD analysis was done by using an X-ray diffractometer with graphite monochromatized Cu-Kα radiation (wavelength = 1.5418 Å) in order to assess the nature of nanoparticles (TiO_2_ and SiO_2_).

#### 2.4.5. Evaluating the Antibacterial Activity of Nanoparticles and Natural Gums

In order to evaluate the antifungal potential of synthesized NPs and gum, a well diffusion bioassay was conducted as described by Rajeshkumar and Malarkodi [35]. Briefly, *B. velezensis* was cultured in an NB medium and incubated at 28 °C. After two days, 100 μL of bacterial suspension was spread on Nutrient Agar (NA) plates. On the medium, 6-mm-diameter wells were punched, and the antibacterial activity of the targeted compounds was assessed by pouring each into a well. Control wells received distilled water. The plates were incubated at 28 °C for 24 h, and the inhibition zone diameters were measured.

#### 2.4.6. Preparation of Bacterial Cell Culture

In order to prepare bacterial cell culture, a pure culture of *B. velezensis* was transferred into a flask containing the NB medium and incubated at 28 °C for 48 h on a rotary shaker at 120 rpm. All materials and equipment used in this research were sterilized prior to being used.

#### 2.4.7. Preparation of Microcapsules

For preparing microcapsules, different compositions of biopolymers, including Alg, BG, MG, GG, ZG, WPC, Alg-BGMG, Alg-GG, Alg-ZG, and Alg-WPC, were used to embed *B. velezensis*. Sodium alginate and whey protein were purchased from the Sigma Company and natural gums were collected manually from forest trees. Encapsulation was conducted according to the method described by He et al. [36]. Briefly, suspensions having different compositions were prepared using sodium alginate (1.5%), MG (1%), GG (1%), ZG (2%) and WPC (8%), TiO_2_ NPs, and SiO_2_ NPs. The suspension was mixed with bacterial strain broth in a 2:1 ratio. The prepared mixtures were added dropwise into a CaCl_2_ crosslinking solution. After 2 h, the synthesized capsules were rinsed thrice with sterile water and dried in an oven at 40 °C. In addition to the layer-by-layer technique for preparing microcapsules, a multilayer technique was employed to encapsulate the studied bacteria. In order to prepare the multilayer microcapsules, Alg-WPC-ZG and Alg- MG-ZG were used. In this technique, the studied bacterium was first encapsulated with alginate and then, in two consecutive steps, the mentioned polymers were used to cover the Alg microcapsules. In this study, the performance of multilayer microcapsules against two-layer microcapsules was assessed as well.

### 2.5. Instrumental Characterization of Microcapsules

#### 2.5.1. SEM Analyses

Scanning electron microscopy (SEM; TESCAN S8000, Brno, Czech Republic) was performed to investigate the topography and geometrical characteristics of the microcapsules. For this purpose, the sample was dried at 40 °C for 24 h and sputter-coated with gold for SEM analysis.

#### 2.5.2. FTIR Analyses

The functional groups involved in the synthesized microcapsules were identified using a Fourier transformation infrared (FTIR) spectrophotometer. Briefly, the Alg, WPC, ZC, Alg-WPC, and Alg-WPC-ZG, microcapsules were powdered by grinding, and the obtained powder was subjected to FTIR measurements. FTIR analyses were conducted by the KBr pellet technique using FTIR equipment (EQUINOX 3000, INEL, Artenay, France). The spectra were obtained over the range from 4000 to 400 cm^−1^ with a resolution of 4 cm^−1^.

#### 2.5.3. XRD Analyses

Alg, ZG, WPC, Alg-WPC, and Alg-WPC-ZG microcapsules without bacteria were powdered by grinding and subjected to XRD analysis. Microcapsules were characterized using an X-ray instrument (Bruker) equipped with CuKa (λ = 1.54 Å) radiation. X-ray diffraction was recorded in the region of 2*θ* from 10° to 80° using an XRD diffractometer (D8-Advance, Bruker, Billerica, MA, USA)

### 2.6. Moisture Content of Microcapsules

In order to check the swelling percentage of the microcapsules, one gram of wet microcapsules was weighed accurately. Again, after drying at 40 °C, the weight of the microcapsules was measured precisely. The moisture content of the microcapsules was calculated using the following equation (Equation (3)) [18]:

(3) $$\mathrm{Moisture}\;\mathrm{content}\;\left(\%\right)=\frac{\mathrm{Weight}\;\mathrm{of}\;\mathrm{wet}\;\mathrm{microcapsules}\;\left(g\right)-\mathrm{Weight}\;\mathrm{of}\;\mathrm{dry}\;\mathrm{microcapsules}\;\left(g\right)\;}{\mathrm{weight}\;\mathrm{of}\;\mathrm{wet}\;\mathrm{microcapsules}\;\left(g\right)}\times 100$$

### 2.7. Swelling Ratio of Microcapsules

One gram of dried microcapsules was placed in a 10 mL test tube, and phosphate buffer (pH = 7.4) was added. The microcapsules were immersed in buffer and removed after 24 h; excess water was completely separated with filter paper. Sensitive scales measured the weight of swelling beads. The swelling ratio of the microcapsules was computed as follows (Equation (4)) [37]:

(4) $$\mathrm{Swelling}\;\mathrm{ratio}\;\left(\%\right)=\frac{\mathrm{weight}\;\mathrm{of}\;\mathrm{the}\;\mathrm{swelling}\;\mathrm{microcapsule}-\mathrm{weight}\;\mathrm{of}\;\mathrm{dried}\;\mathrm{microspheres}}{\mathrm{weight}\;\mathrm{of}\;\mathrm{dried}\;\mathrm{microspheres}}\times 100$$

### 2.8. Encapsulation Efficiency

The encapsulation efficiency was assessed as the number of bacteria embedded in the microcapsules. One g of each microcapsule type was added to 10 mL of phosphate buffer and vortexed for 20 min. After preparing the serial dilution, culturing on NA medium was carried out. The encapsulation efficiency of the bacterial formulation was calculated using the following formula (Equation (5)) [18]:

(5) $$\mathrm{Encapsulation}\;\mathrm{efficiency}\;\left(\%\right)=\frac{\mathrm{Bacteria}\;\mathrm{in}\;\mathrm{the}\;\mathrm{microcapsules}\;\left(\mathrm{CFU}\frac{g}{1}\right)\times 100}{\mathrm{Bacteria}\;\mathrm{added}\;\mathrm{to}\;\mathrm{formulation}\;\left(\mathrm{CFU}\frac{g}{l}\right)}$$

### 2.9. Evaluation of Bacterial Release from Microcapsules

A dialysis bag technique was used to investigate the release of bacteria from the microcapsules [38]. For this test, one gram of Alg-ZG, Alg-WPC, and Alg-WPC-ZG microcapsules was weighed, and, after pouring into the dialysis bag, its surroundings were completely sealed. Then, it was placed in one liter of phosphate buffer (pH = 7.4) and maintained at room temperature. At certain time points (5, 10, 15, 20, 25, 30, 35, 40, 45, and 50 days), after preparing the dilution series, the population of bacteria released from the microcapsules was counted.

### 2.10. Statistical Analysis

One-way ANOVA analyzed the data on bacterial release, viability, and growth factors. Significant SAS 9.1 (SAS Institute, Inc., Cary, NC, USA) and Mean separation was accomplished using the Tukey test, *p* = 0.05 Origin v. 8.0 software (OriginLab Corporation, Northampton, MA, USA) was used for data analysis, drawing XRD patterns.

## 3. Results

### 3.1. In Vitro Evaluation of the Antifungal Activity of Bacillus velezensis against Gaeumannomyces graminis var. tritici

**Figure 1:**
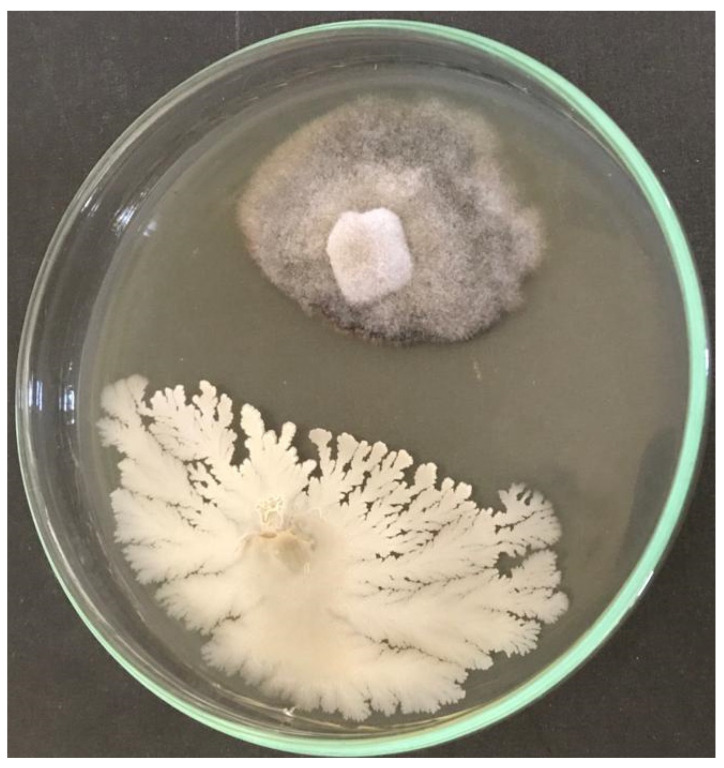
In vitro antifungal activity of *Bacillus velezensis* against *Gaeumannomyces graminis* var. *tritici*.

The assessment of in vitro antifungal activity of *B. velezensis* reveals that this antagonist has antifungal potentiality in suppressing *G. gramminis* by forming a significant inhibition zone (Figure 1).

### 3.2. Evaluation of Bacillus velezensis to Produce Some Metabolites

**Figure 2:**
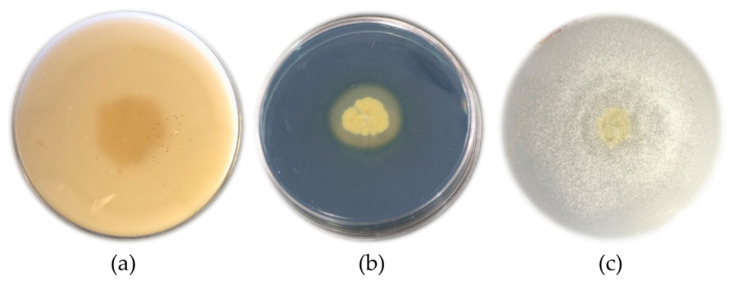
The ability of *Bacillus velezensis* to produce some metabolites. (**a**) The positive proteolytic activity of *B. velezensis* shows a clear halo around the bacterial colony; (**b**) the positive activity of *B. velezensis* in the production of siderophore changes the color of the medium to orange; (**c**) the positive activity of *B. velezensis* dissolves phosphate as seen by the presence of a colorless halo around the colony.

Results revealed that the antagonistic Bacillus could produce protease, lipase, indol, and siderophore metabolites. *B. velezensis* was also able to dissolve mineral phosphate. Specifically, proteolytic activity was demonstrated by forming a clear halo around the bacterial colony. The protease activity of the target bacterium was positive, with a halo diameter of 26 mm (Figure 2a). The results of the lipase production test showed that the desired strain could produce a lipase enzyme, which was determined by the formation of sediments around the colony. Based on the study on the auxin production by *B. velezensis* in a TSB medium containing L-tryptophan, it was observed that the studied strain could produce indole acetic acid, and its production yield was recorded as 1.96 µg/mL. According to Kloepper et al. [39], rhizospheric bacteria stimulating plant growth can modify root architecture by producing the auxin hormone. Siderophore production was determined by changing the color of the medium from blue to orange in the CAS agar medium. The changing color of the medium to orange proved the ability of *B. velezensis* to produce siderophore (Figure 2b). The results of the strain-bacterial phosphate dissolution in the presence of a colorless halo around the colony showed that this bacterium was able to dissolve phosphate (Figure 2c).

### 3.3. Characterization of SiO_2_ and TiO_2_ NPs

**Figure 3:**
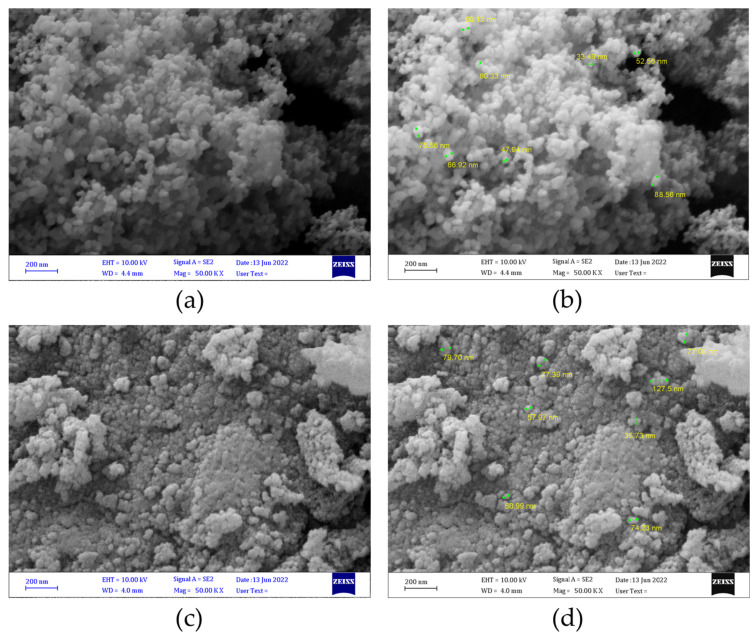
Scanning electron micrographs of TiO_2_ NPs (**a**,**b**) and SiO_2_ NPs (**c**,**d**). (**b**) and (**d**) display measurements of TiO_2_ NPs and SiO_2_ NPs, respectively.

SEM micrographs confirmed the successful synthesis of NPs. SEM images showed the SiO_2_ and TiO_2_ NPs have an approximately spherical shape with a size around 50–100 nm. Figure 3a represents the SEM micrograph of TiO_2_ NPs and Figure 3b represents the SEM micrograph of SiO_2_ NPs.

**Figure 4:**
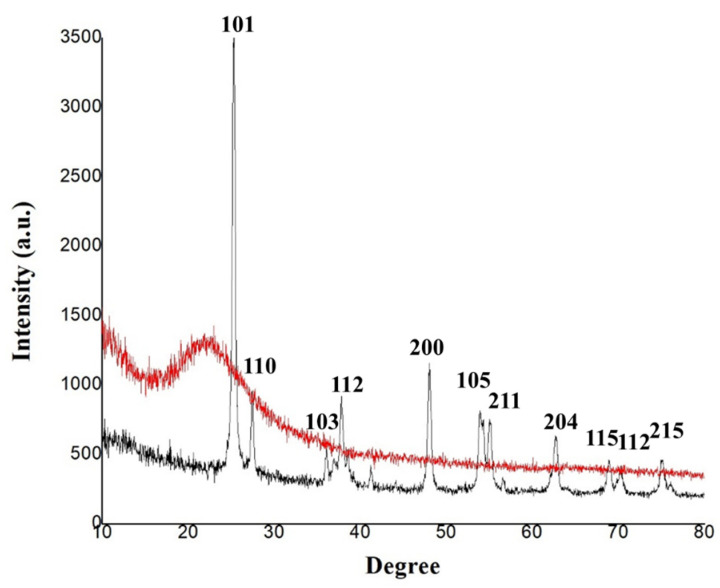
The XRD spectra of SiO_2_ (red pattern) and TiO_2_ NPs (black pattern).

In the XRD analysis of silica NPs, the characteristic amorphous band of SiO_2_ NPs was revealed without any sign of crystallization, which is in accordance with the results reported by Zulfiqar et al. [40]. Figure 4 showed the XRD pattern of the synthesized TiO_2_ NPs. The XRD pattern is consistent with that reported for TiO_2_ NPs by other researchers [41]. The peaks at 2*θ* =25.40° and 2*θ* = 48.01° confirm the TiO_2_ anatase structure [40,42]; in fact, the presence of this couple of strong diffraction peaks is a clear indicator of the anatase phase [10]. No spurious diffraction peaks were observed in the sample; according to the results of Varshney et al. [43], the lack of spurious diffractions demonstrates the crystallographic purity of the sample analyzed [43].

### 3.4. Evaluating the Antibacterial Activity of Nanoparticles and Natural Gums

Before developing the microcapsule formulation, it was crucial to examine the effects of nanoparticles and gums employed in the formulation on the viability of the researched bacterial strain. For this purpose, the well diffusion method was used. After 48–72 h, no halo of bacterial growth around the well was observed, which shows that these nanoparticles and gums have no inhibitory effect on bacterial growth in this study.

### 3.5. Instrumental Characterization of Microcapsules

#### 3.5.1. SEM Analyses

**Figure 5:**
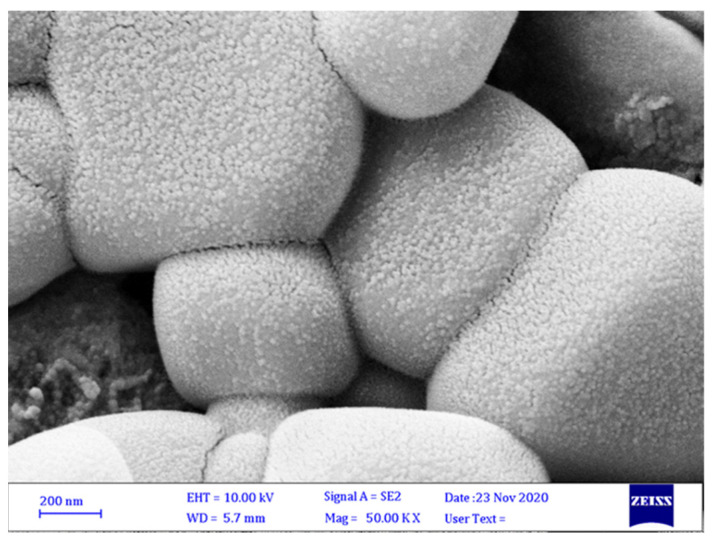
Scanning electron micrograph of microcapsules. SEM revealed that most microcapsules were cubic with quite homogeneous morphology.

Scanning electron microscopy analysis confirmed the formation of microcapsules. Figure 5 shows the surface morphology of Alg-WPC-ZG, and the entire microcapsules can be seen. SEM revealed that most microcapsules were cubic with quite homogeneous morphology.

#### 3.5.2. FTIR Analyses

**Figure 6:**
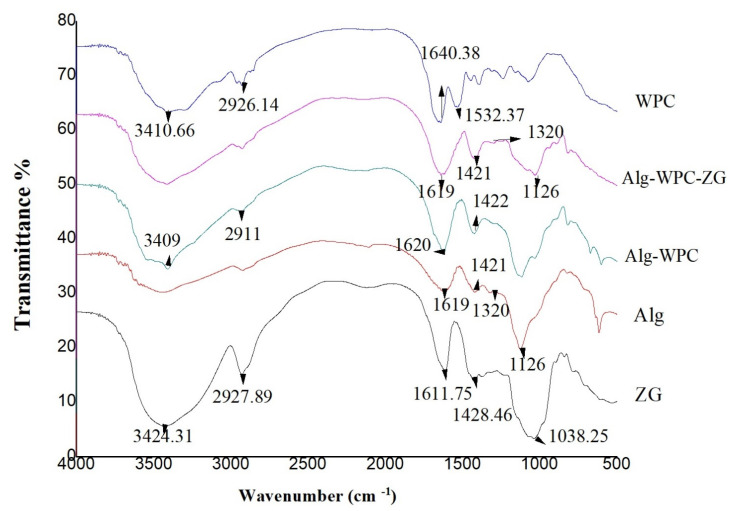
FTIR spectra of alginate (Alg), whey protein (WPC), zedo gum (ZG), alginate-whey protein microcapsules (Alg-WPC), and alginate-whey protein-zedo gum microcapsules (Alg-WPC-ZG).

In general, the FTIR spectra of the hydrocolloid materials showed a specific peak of O–H stretching and intra- and intermolecular hydrogen bonds between 3650 cm^−1^ and 3000 cm^−1^, being a wide band. Another peak in the region between 2950 cm^−1^ and 2800 cm^−1^ can be related to C–H stretching vibrations. These two bands are characteristics of all polysaccharides. FTIR peaks of alginate, zedo gum and whey protein, alginate-zedo gum, and Alg-WPC-ZG have been represented in Figure 6. In the FTIR spectra of ZG, the peaks at approximately 3424 cm^−1^ are related to the stretching vibration modes of O=H bound to carbons, and the peaks of asymmetric –CH_2_– functional groups were observed at 2927 cm^−1^. The peak at 1423 cm^−1^ for ZG is related to carboxylate groups which are responsible for the negative charge of the biopolymer (Figure 6) [21]. The FTIR spectra of alginate showed two specific bands at 1619 and 1421 cm^−1^ related to a carbonyl group of carboxylic acid [44] and asymmetric and symmetric stretching vibrations of the C–O bond of the carboxylate salt ion [45]. Additionally, the spectral peak at 1320 cm^−1^ suggests the presence of guluronic acid, the band at 1126 cm^−1^ being related to the C–O stretching vibrations of the pyranosyl ring (Figure 6) [46]. The FTIR spectrum of WPC is confirmed by the band at 1532 cm^−1^ related to the amide ΙΙ band which is related to the aggregation of intermolecular β-sheets and peak at 1640 cm^−1^ due to C=O stretching of the amide Ι band, which confirms the attendance of antiparallel β-sheets (Figure 6) [47]. The FTIR peaks of the three components are retained in the encapsulation process. Bands of amide Ι (at 1638 cm^−1^ in WPC) and asymmetric stretching of carboxyl (at 1619 cm^−1^ in Alg) overlapped. A broad and strong absorption band appears at 3600–3200 cm^−1^ in the three polymers, representing the stretching vibration of the intermolecular –OH group, which possibly plays a basic role in the formation of microcapsules (Figure 6).

#### 3.5.3. XRD Analyses

**Figure 7:**
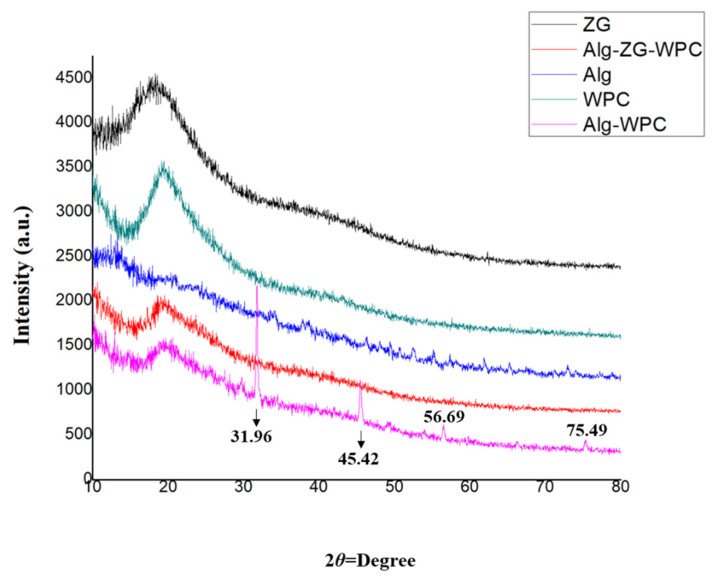
The XRD spectra of different biopolymers (zedo gum, alginate, and whey protein), alginate-zedo gum-whey protein, and alginate-whey protein microcapsules.

The XRD spectra of the synthesized microcapsules using zedo gum, alginate, whey protein, alginate-zedo gum-whey protein, and alginate-whey protein are depicted in Figure 7. The crystalline nature of the components and the microcapsule was determined by analyzing the XRD patterns. In the XRD spectrum of WPC, a peak at 2*θ* = 10° and 2*θ* = 20° was observed. An X-ray diffractogram of Alg affirms the crystalline nature of the alginate, based on the emission peak at 2*θ* value at 10°. In the Alg-WPC microcapsules, the sharp peaks of Alg and WPC completely disappeared; therefore, it can be concluded that the Alg-WPC microcapsules were synthesized with an amorphous structure. This structure can result from the electrostatic interaction between the carboxyl group of alginate and the amino group of whey proteins. It has been reported that amorphous natures are more soluble, so they release the entrapped cells more readily [48]. A rather sharp peak at 2*θ* = 20° indicated the semi-crystalline microstructure of the ZG. In the Alg-WPC-ZG composition, the basic structures of the component materials seem to be preserved.

### 3.6. Moisture Content and Swelling Ratio of Microcapsules

According to ANOVA results, the moisture content and swelling ratio of microcapsules showed that the effect of treatments was significant (*p* < 0.01). Comparing the average percentage of moisture and swelling showed a significant difference between the microcapsules. Moisture content in Alg-WPC-ZG, Alg-ZG, and Alg-WPC microcapsules was 75.82%, 74.26%, and 68.33%, respectively, and the swelling ratio was 123.33%, 118.66%, and 116.66%, respectively (Table 1).

### 3.7. Encapsulation Efficiency

Encapsulation efficiency is one of the essential factors affecting the performance of encapsulation systems [49]. The encapsulation of *B. velezensis* by layer-by-layer and multilayer methods using different combinations resulted in high encapsulation efficiency (>67%), as shown in Table 1. The encapsulation efficiencies of the microcapsules were 94.33%, 92.33%, and 91% for Alg-WPC-ZG, Alg-ZG, and Alg-WPC, respectively. The results revealed that the mixture of Alg-WPC-ZG offers the highest encapsulation efficiency due to providing a multilayer coating of the wall materials around the bacterial cells. Table 1 presents the encapsulation efficiency with the different polymers studied.

### 3.8. Evaluation of Bacterial Release from Microcapsules

**Figure 8:**
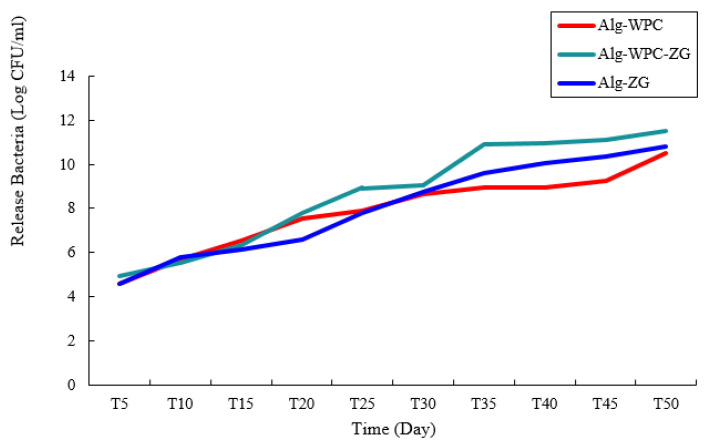
Release of *B. velrzensis* from microcapsules in phosphate buffer over 50 days.

The effect of different formulations (Alg-ZG, Alg-WPC-ZG, and Alg-WPC) on the release of bacterial strain during storage at 28° C for 0–50 days are shown in Figure 8. The number of released bacteria from microcapsules was determined by the plate count method on NA medium plates [25]. The number of bacteria released from the three microcapsules increased rapidly until the 50th day after the preparation of the formulation. The maximum number of viable cells was recorded on the 50th day, released from Alg-WPC-ZG microcapsules.

## 4. Discussion

Plant pathogens with a long history cause a wide range of harm to plants, including disruption of physiological function, imbalance in secondary metabolite synthesis, and a reduction in crop yield. Using conventional formulations of pesticides to combat plant pathogens has a detrimental side effect on living organisms and the environment and causes extended resistance of many plant pathogens to these agrochemicals. Using antagonist bacteria as biological control agents is a valuable alternative to the chemical control of plant diseases. Many studies have reported the efficacy of the Bacillus species [50], fluorescent Pseudomonas [51], and Streptomyces species [2] in controlling many plant pathogens. These microorganisms are the best option for achieving sustainable agriculture. Rapid and successful laboratory identification is required for the environmental application of these biocontrol bacteria. The zone of inhibition method is a well-known and rapid method for the initial selection of antagonist bacteria against pathogens. It is believed that isolates with a high ability to control pathogens in vitro have unique properties that can control them under in vivo conditions [52]. Antagonist bacteria have several indirect and direct mechanisms to enhance plant growth and control disease. Auxin has a significant role in increasing the surface area of roots and the number of root hairs, increasing nutrient uptake, and stabilizing the plant in the soil, thereby improving plant growth and yield [53]. The production of extracellular enzymes such as proteases and lipases is essential in controlling plant pathogens. Proteins and lipids are essential components of the cell wall of many pathogens so extracellular enzymes can break down pathogen cells [54]. Phosphorus (*p*) is a vital nutrient in the plant that helps essential functions such as macromolecule biosynthesis, respiration, energy conversion, and plant growth. Because most *p* is insoluble in soil, it is not absorbable by plants. Therefore, phosphorus solubilizing bacteria can be helpful in plant phosphorus uptake. These bacteria can dissolve mineral phosphate by releasing organic acids and lowering the pH of the environment [55]. One of the most significant plant hormones, Indole-3-acetic acid (IAA), is involved in many plant functions, including photosynthesis and respiration. IAA-producing bacteria play an essential role in plant growth and its development. Auxin is one of the influential factors in increasing the number of lethal fibers and the lateral root density [56]. Biological agents in commercial formulations have poor quality formulation and low stability. These formulations have low efficacy due to the non-targeted delivery of bioactive substances to the target [57,58,59,60,61]. Therefore, the search for more influential formulations is one of the main emphases of sustainable agriculture. Encapsulation efficiency is related to the method used for encapsulation. The layer-by-layer technique involves gentle processes that lead to the relatively high survival of bacterial cells [62]. Vejan et al. [4] indicated that the encapsulation efficiency of alginate and brown-rice protein microcapsules of *Bacillus salmalaya* 139S1 was 99.7%. Regarding microcapsules, the moisture content and swelling ratio are essential parameters in determining the amount of water absorbed and lost from microcapsules. These parameters play an essential role in releasing bacterial cells into the soil. When there is water in the soil, the microcapsules absorb the water and release the bacteria they have been holding. When the water level drops, the bacteria are no longer released [16]. Surface morphology is one of the most important factors in determining the release behavior of the internal agent from the microcapsule. Surface interactions of polymers can alter the surface morphology and affect the release rate. The smooth surface of the microcapsules indicates that the main material inside the capsules is retained, resulting in higher microencapsulation efficiency. The rhizosphere is a very nutrient-rich environment in which soil microorganisms compete fiercely for their energy resources and to obtain the appropriate ecological niche for habitat. Although biocontrol mechanisms are complex and diverse, in order to achieve successful biocontrol, biocontrol agents must be present at the right time and place. This indicates the importance of colonization [63]. Therefore, the use of compounds that can increase the colonization of plant roots has an important role in the formulation of biological agents. Saberi-Rise and Moradi-Pour [15] showed that titania nanoparticles increased the percentage of bacillus colonization of bean roots. Silica nanoparticles have shown a high ability to increase plant performance against environmental stresses [64]. Therefore, the encapsulation of biocontrol bacteria with nanoparticles simultaneously eliminates the essential needs of the plant and increases the performance of the encapsulated agents. It is expected that the use of this formulation can have a high performance in the future by controlling plant pathogens and increasing the growth of agricultural products.

As our results indicate, encapsulated biological control agents can be considered a promising formulation for the influential management of plant pathogens in the future. In order to make this wish a reality, more research is required to gain better insight into understanding the physiology and environmental achievements of these formulations, including their positive or negative effects on the stability of the soil, their relation with other plant pathogens, and their relation with other biological control agents in the soil.

## 5. Conclusions

Biological control agents are an essential part of sustainable agriculture and contribute to controlling plant pathogens. Formulations based on biological control agents or their bioactive metabolites are very few and often ineffective. The conventional chemical methods used to suppress plant pathogens cause environmental challenges. The results presented in this research showed that the new multi-layer Alg-WPC-ZG formulation enriched with TiO_2_/SiO_2_ NPs was effectively able to encapsulate and release Bacillus velezensis bacteria (encapsulation efficiency = 94.3%). Therefore, it is expected that, with practical use in soil conditions, it can perform a better function in regulating the population of bacteria released from the microcapsule, thus controlling plant pathogens. The microcapsules protect the bacteria against environmental factors and can be applied for loading biological agents against biotic stresses. Therefore, the use of this formation may constitute a new step in the biological management of plant pathogens in the future.

## Figures and Tables

**Table 1:** The results of the evaluation of moisture content, swelling ratio, and encapsulation efficiency of *B. velezensis* microcapsules prepared by different polymers.

Treatments	Swelling Ratio %	Moisture Content %	Encapsulation Efficiency %
Alg-ZG-WPC	123.333 ± 1.452 ^a^	75.820 ± 0.320 ^a^	94.333 ± 0.881 ^a^
Alg-ZG	118.667 ± 0.881 ^ab^	70.246 ± 0.301 ^b^	92.333 ± 0.333 ^a^
Alg-WPC	116.667 ± 0.881 ^ab^	68.330 ± 0.766 ^bc^	91.000 ± 0.577 ^a^
Alg-MG-ZG	113.000 ± 1.527 ^b^	66.120 ± 0.069 ^c^	85.667 ± 0.881 ^b^
Alg-MG	105.667 ± 2.603 ^c^	57.380 ± 0.643 ^d^	70.667 ± 1.201 ^c^
Alg-TG	101.667 ± 0.666 ^c^	54.176 ± 0.595 ^e^	67.667 ± 1.452 ^c^

For a given parameter, means with the different letter(s) in each column are significantly different (Tukey test, *p* = 0.05). Alginate (Alg), whey protein (WPC), zedo gum (ZG), mastic gum (MG), and tragacanth gum (TG).
